# System accuracy evaluation of 18 CE-marked current-generation blood glucose monitoring systems based on EN ISO 15197:2015

**DOI:** 10.1136/bmjdrc-2019-001067

**Published:** 2020-01-15

**Authors:** Stefan Pleus, Annette Baumstark, Nina Jendrike, Jochen Mende, Manuela Link, Eva Zschornack, Cornelia Haug, Guido Freckmann

**Affiliations:** Institut für Diabetes-Technologie Forschungs- und Entwicklungsgesellschaft mbH, Universität Ulm, Ulm, Germany

**Keywords:** accuracy, blood glucose monitors, blood glucose self-monitoring, health care research

## Abstract

**Objective:**

Accuracy of 18 current-generation blood glucose monitoring systems (BGMS) available in Europe was evaluated applying criteria adapted from EN ISO 15197:2015 with one reagent system lot. BGMS were selected based on market research data.

**Research design and methods:**

The BGMS ABRA, Accu-Chek Guide, AURUM, CareSens Dual, CERA-CHEK 1CODE, ContourNext One, eBsensor, FreeStyle Freedom Lite, GL50 evo, GlucoCheck GOLD, GlucoMen areo 2K, GluNEO, MyStar DoseCoach, OneTouch Verio Flex, Pic GlucoTest, Rightest GM700S, TRUEyou, and WaveSense JAZZ Wireless were tested using capillary blood from 100 different subjects and assessing the percentage of results within ±15 mg/dL (0.83 mmol/L) or 15% of comparison method results for BG concentrations below or above 100 mg/dL (5.55 mmol/L), respectively. In addition, the minimal deviation from comparison method results within which ≥95% of results of the respective BGMS were found was calculated.

**Results:**

In total, 14 BGMS had ≥95% of results within ±15 mg/dL (0.83 mmol/L) or ±15% and 3 BGMS had ≥95% of results within ±10 mg/dL (0.55 mmol/L) or ±10% of the results obtained with the comparison method. The smallest deviation from comparison method results within which ≥95% of results were found was ±7.7 mg/dL (0.43 mmol/L) or ±7.7%; the highest deviation was ±19.7 mg/dL (1.09 mmol/L) or ±19.7%.

**Conclusions:**

This accuracy evaluation shows that not all CE-labeled BGMS fulfill accuracy requirements of ISO 15197 reliably and that there is considerable variation even among BGMS fulfilling these criteria. This safety-related information should be taken into account by patients and healthcare professionals when making therapy decisions.

**Trial registration number:**

NCT03737188.

**Significance of this study**

**What is already known about this subject?**

- Tight glycemic control is an essential prerequisite for people suffering from diabetes mellitus to avoid dangerous metabolic states (hypoglycemia or hyperglycemia).
- Clinical value of blood glucose monitoring systems (BGMS) is associated with measurement accuracy.
- Studies within recent years have shown that there is a considerable number of CE-labeled BGMS on the market which do not fulfill ISO requirements reliably.

**What are the new findings?**

- Although only current-generation BGMS were tested, more than 20% did not meet ISO 15197 accuracy criteria with their tested reagent system lot.
- Considerable variation in measurement accuracy was found even among BGMS that meet these criteria.
- Regular post-market surveillance of all available BGMS is necessary to allow patients with diabetes and healthcare professionals selecting a BGMS that is best possible for diabetes therapy.

**How might these results change the focus of research or clinical practice?**

- These results should be taken into account by patients and healthcare professionals when choosing a BGMS for therapy or when making therapy decisions.

## Introduction

Systems for self-monitoring of blood glucose are widely perceived as an essential tool for people suffering from diabetes mellitus enabling tight glycemic control and therefore supporting adequate clinical decisions made by caregivers and patients.^1–4^ The use of blood glucose monitoring systems (BGMS) for therapy decisions has the potential to prevent late complications^4 5^ and facilitates insulin adjustment especially in patients with multiple daily injections or insulin pumps but is of therapeutic benefit for all people with diabetes in general.^1 4 6 7^ In this context, accurate blood glucose (BG) measurement results are an essential prerequisite as they help to avoid dangerous hypoglycemic and hyperglycemic metabolic conditions.

Nowadays, a broad variety of BGMS is available on the market and it is understood that clinical value is associated with measurement accuracy. Thus, healthcare professionals and patients need points of reference when choosing between systems assignable to different price ranges and to different technological stages.^8–10^

The International Organization for Standardization (ISO) standard 15197, which was first published in 2003 and revised with more stringent system accuracy criteria in 2013^11^ (harmonized as EN ISO 15197:2015), defines accuracy requirements for BGMS.

Manufacturers often apply the ISO 15197 standard to obtain the Conformité Européenne (CE) mark for their system, which is a minimum requirement for a product to be marketed in the European Union. Nevertheless, several evaluation or surveillance studies within recent years repeatedly revealed that a non-negligible number of CE-marked BGMS, which do not fulfill the minimum accuracy criteria of ISO 15197, had been or currently still are on sale on the European market.^12–14^

Regarding system accuracy, the minimal demands of ISO 15197 are at least 95% of a system’s measurement results shall be within ±15 mg/dL (0.83 mmol/L) of the reference measurement procedure’s results at BG concentrations <100 mg/dL (5.55 mmol/L) and within 15% at BG concentrations ≥100 mg/dL (5.55 mmol/L) (system accuracy criterion A) and at least 99% of results shall be within the clinically acceptable zones A and B of the consensus error grid (CEG) (system accuracy criterion B). Compliance with system accuracy criteria must be shown for three different reagent system lots.^11^

However, at present, there is no harmonization of reference measurement procedures for evaluating BGMS accuracy and current BGMS are typically calibrated against either a glucose oxidase (GOD)–based or a hexokinase (HK)-based reference measurement procedure.^15^

This study’s aim was the evaluation of 18 different, current-generation BGMS which are currently available on the European market with one reagent system lot each based on test procedures defined in ISO 15197 with their respective manufacturer’s reference measurement procedure. The study was financially supported by unrestricted grants from six different BGMS manufacturers which had no impact on study procedures and BGMS acquisition.

## Research design and methods

This surveillance study of BGMS accuracy was conducted in November and December 2018 at the Institut für Diabetes-Technologie Forschungs- und Entwicklungsgesellschaft mbH an der Universität Ulm (IDT), Ulm, Germany, in compliance with the German Medical Devices Act, the Guideline for Good Clinical Practice, and under consideration of the Declaration of Helsinki (revised edition, Fortaleza 2013). Experimental procedures were performed by trained study personnel based on the requirements described in detail in ISO 15197, clause 6.3.

### Study population

To obtain 100 evaluable data sets as required by ISO 15197, 126 subjects were included comprising 44 subjects with type 1 diabetes, 58 subjects with type 2 diabetes, and 24 subjects not having diabetes at the time of study conduct. The participants’ age ranged from 20 to 82 years. Mean age of all participants was 59.4±14.0 (mean±SD) years. Participants were examined by a physician to check eligibility after they signed the informed consent form. In that respect, the subjects’ anamnesis, including medication and interfering substances (eg, acetaminophen, salicylates, ascorbic acid, dopamine) indicated in the respective manufacturer’s labeling, and inclusion and exclusion criteria for study participation (exclusion criteria for example, pregnancy, lactation period, severe acute disease, severe chronic disease, legal incompetence, compromising mental constitution) were checked.

### Blood glucose monitoring systems

In this study, 18 different CE-marked BGMS were evaluated with one reagent system lot each (table 1, online supplementary table S1). BGMS were selected based on volume of sold reagent system units in 08/2017 provided by a market research institute (IMS MIDAS Customised Insights, analysis period MAT 08/2017). In order to cover a wide variety of manufacturers, volumes of sold reagent system units were grouped across all reagent systems of individual manufacturers/distributors. For each of the 18 manufacturers/distributors with the highest volume of sold reagent system units, a current-generation BGMS was selected. The system selection was carried out with the aim of giving a comprehensive overview of market-approved current-generation BGMS. Meters and reagent system lots were independently purchased on the market by the investigator (IDT) from pharmacies across Europe in the Czech Republic, France, Germany, Greece, Italy, the Netherlands, Poland, and the UK. The systems were adjusted, stored, and used in compliance with the respective manufacturer’s labeling. The proper functioning of each system was ensured at least once a day with control measurements according to the manufacturer’s instructions prior to the test procedures. Meter Trax control solutions (Bio-Rad Laboratories, Irvine, CA, USA) were used for eBsensor and Pic Gluco Test because proprietary control solutions were not available, whereas BGMS-specific control solutions were used with all other BGMS. Except for eBsensor, which is a whole blood–calibrated BGMS, all systems displayed plasma-equivalent glucose concentrations. Therefore, results of the applied reference measurement procedure were converted to whole blood readings for this system’s further evaluation (see section “*Reference measurement procedures”*). The BGMS CareSens Dual, GluNEO, and WaveSense JAZZ Wireless provided blood glucose results in millimoles per liter that were converted to milligrams per deciliter by using the following formula: 1 mmol/L=18.02 mg/dL. All other systems displayed glucose results in milligrams per deciliter.

10.1136/bmjdrc-2019-001067.supp1 Supplementary data

**Table 1:** System characteristics according to the respective manufacturer’s labeling for blood glucose monitoring systems (BGMS) a to r

#	System	Reagent system		Manufacturer’s reference measurement procedure	Test strip enzyme	Manufacturer*
		Lot number	Expiry date			
a	ABRA	MPD123C006	2020/03	GOD	GOD	Diagnosis S.A., Poland
b	Accu-Chek Guide	100551	2019/10	HK	GDH	Roche Diabetes Care GmbH, Germany
c	AURUM	TD16G115-BEE	2019/01	GOD	GDH	TaiDoc Technology Corp., Taiwan
d	CareSens Dual	QM20HBB2B	2020/04	GOD	GDH	i-SENS, Inc., Korea
e	CERA-CHEK 1CODE	G48D071711	2020/04	GOD	GDH	Green Cross Medis Corp., Korea
f	ContourNext One	DP8APEG14B	2020/01	GOD	GDH	Ascensia Diabetes Care Holdings AG, Switzerland
g	eBsensor	I2A0B1H05	2020/02	GOD	GOD	Visgeneer Inc., Taiwan
h	FreeStyle Freedom Lite	1041095	2020/02	GOD	GDH	Abbott Diabetes Care Ltd., UK
i	GL50 evo	D07/1	2020/02	GOD	GDH	Beurer GmbH, Germany
j	GlucoCheck GOLD	WG18A103-BEE	2020/01	GOD	GDH	aktivmed GmbH, Germany
k	GlucoMen areo 2K	HS180320	2020/03	GOD	GOD	A.Menarini Diagnostics S.r.l., Italy
l	GluNEO	X18C17-5B2	2020/03	HK†	GDH	Infopia Co., Ltd., Korea
m	MyStar DoseCoach	PM15WD96L	2019/01	GOD	GOD	AgaMatrix Inc., USA Distributor‡: Sanofi-Aventis France, France
n	OneTouch Verio Flex	4341526	2019/08	GOD	GDH	LifeScan Europe, Division of Cilag GmbH International, Switzerland
o	Pic Gluco Test	1018050006	2020/05	GOD	GOD	SD BioSensor, Inc., Korea
p	Rightest GM700S	2117A1201	2019/09	GOD	GDH	Bionime Corp., Taiwan
q	TRUEyou	HLU1243INT	2020/10	GOD	GDH	Trividia Health, Inc., USA
r	WaveSense JAZZ Wireless	QA03WY28L	2020/02	GOD	GOD	AgaMatrix Inc., USA

*Manufacturer names are given according to the imprints on the systems.

†No information about the manufacturer’s reference measurement procedure was available at the time of manuscript submission. Based on literature research^19^ and the investigator’s experience regarding reliability of measurement results, the HK-based procedure was assigned as primary reference measurement procedure for system accuracy evaluation.

‡Data from the market research institute (IMS MIDAS Customised Insights, analysis period MAT 08/2017) indicated Sanofi as manufacturer of the reagent system used with BGMS m, as opposed to the labels on the BGMS components that indicate AgaMatrix as manufacturer. This discrepancy was only realized after all materials had been procured; therefore, this BGMS was not replaced.

GDH, glucose dehydrogenase; GOD, glucose oxidase; HK, hexokinase.

### Reference measurement procedures

Reference measurements were performed for each test system with a GOD-based procedure (YSI 2300 STAT Plus glucose analyzer; YSI Incorporated, Yellow Springs, OH, USA) and a HK-based procedure (Cobas Integra 400 Plus; Roche Instrument Center, Rotkreuz, Switzerland) whose output unit was milligrams per deciliter. Conformity to traceability requirements of ISO 17511^16^ of both reference measurement procedures was assured by the respective analyzer’s manufacturer. As required by Rili-BÄK,^17^ the Guideline of the German Medical Association on Quality Assurance in Medical Laboratory Examinations, verification of trueness and precision of both reference measurement procedures was performed during the experimental phase by regular internal and external quality control measurements. In addition, daily quality control measurements following IDT-internal standard operating procedures were performed using higher-order control materials (NIST SRM 965b (National Institute of Standards and Technology, Gaithersburg, MD, USA)). Using the four available glucose concentration levels, the GOD-based and the HK-based methods exhibited biases of ≤2.2% and ≤1.7%, respectively, and coefficients of variation of ≤1.9% and ≤1.3%, respectively. All reference measurements were performed in duplicate with both procedures on capillary plasma samples (see section “*Samples and test procedure*”).

For the whole blood–calibrated system eBsensor, results from both reference measurement procedures were converted from plasma BG values to whole blood–equivalent BG values using the following formula: whole blood BG value (mg/dL)=plasma BG value (mg/dL)×(1–(0.0024×hematocrit value (%)).^18^

The respective manufacturer’s reference measurement procedure for accuracy evaluation as specified in the manufacturer’s labeling is shown in table 1. For GluNEO (system l), no information about the reference measurement procedure and/or the method used for calibration were documented in the manufacturer’s labeling and the manufacturer did not respond to an inquiry made in late November 2018. Therefore, we decided to consider the HK-based procedure as the primary reference measurement procedure for GluNEO. Jeanny and Hope^19^ also used a HK-based procedure for the evaluation of system accuracy of GluNEO without quoting information about the manufacturer’s reference measurement procedure.^19^

### Samples and test procedure

ISO 15197 specifies that the BG concentrations of at least 100 different blood samples originating from different subjects shall be distributed as follows: 5% ≤50 mg/dL (2.77 mmol/L), 15% >50 mg/dL (2.77 mmol/L) to 80 mg/dL (4.44 mmol/L), 20% >80 mg/dL (4.44 mmol/L) to 120 mg/dL (6.66 mmol/L), 30% >120 mg/dL (6.66 mmol/L) to 200 mg/dL (11.10 mmol/L), 15% >200 mg/dL (11.10 mmol/L) to 300 mg/dL (16.65 mmol/L), 10% >300 mg/dL (16.65 mmol/L) to 400 mg/dL (22.20 mmol/L), and 5% >400 mg/dL (22.20 mmol/L).^11^ Regarding each reference measurement procedure, blood samples were distributed into the different concentration categories based on the values of the respective procedure. To verify sample stability, the drift between mean values of consecutive duplicate reference measurements must not have exceeded ±4 mg/dL (0.22 mmol/L) at BG concentrations <100 mg/dL (5.55 mmol/L) and ±4% at BG concentrations ≥100 mg/dL (5.55 mmol/L).

Prior to performing any study procedures, participants were asked to wash and dry their hands. Subsequently, the BG measurements were performed in a laboratory setting with controlled room temperature (21.0°C to 24.1°C) and controlled relative humidity (32.4% to 50.9%) in compliance with the manufacturers’ specifications and ISO 15197. The experimental procedures were performed by study personnel trained to the limitations of the BGMS, the manufacturers’ labelings, the safety practices, and the test protocol.

Measurements were performed in duplicate on an individual sample with two different meters of each BGMS using test strips from the same package or vial. Test strips were taken from at least 10 different packages or vials which were changed after approximately 10 subjects.

For BG concentrations >50 mg/dL (2.77 mmol/L) to ≤400 mg/dL (22.20 mmol/L), only unaltered samples were used. The measurement procedure for these samples was as follows: Study personnel collected fresh capillary blood samples in lithium heparin tubes from the participants’ fingertips by skin puncture for duplicate reference measurements. BG concentration was measured with BG meters 1 and 2 of the respective BGMS directly from the puncture site. After that, a second sample for duplicate reference measurements was collected. The order in which BGMS were used was rotated between subjects.

ISO 15197 allows adjustment of glucose concentrations ≤50 mg/dL (2.77 mmol/L) and >400 mg/dL (22.20 mmol/L), if insufficient numbers of unaltered samples are achieved. Additional samples with BG concentrations ≤50 mg/dL (2.77 mmol/L) were prepared as follows: the blood samples were collected in lithium heparin tubes, incubated at room temperature to allow for glycolysis, and gently mixed before testing. Additional samples with BG concentrations >400 mg/dL (22.20 mmol/L) were prepared as follows: the blood samples were collected in lithium heparin tubes, supplemented with concentrated glucose solution (2.22 mol/L glucose in 154 mmol/L NaCl), and gently mixed before testing. Samples with adjusted glucose concentrations were applied to the BGMS with a syringe.

In adjusted samples, the PO_2_ was checked by using a blood gas analyzer (Opti Check; OPTI Medical Systems Incorporation, Roswell, GA, USA) immediately after the test procedure to ensure that their PO_2_ values are comparable with levels found in native capillary blood samples.^20 21^

Additional capillary blood samples were obtained for the determination of hematocrit values which had to be within the range specified in the BGMS’ labelings. For this purpose, samples were collected in heparinized capillaries, the capillaries were centrifuged, and the hematocrit values were determined with the help of an alignment chart traceable to a calibrated ruler.

Samples collected for reference measurements were centrifuged within 10 min of collection to obtain plasma. Plasma was separated immediately after centrifugation. Samples were measured within approximately 30 min of separation (median time to measurement approximately 10 min), so that possible sample degradation should only have negligible effects. Before the measurements with each BG meter and before each aliquot collection for reference measurements, a fresh blood drop was generated (residual blood was wiped off the finger or the syringe beforehand).

### Statistical analysis

For each system, 200 data points from at least 100 capillary samples from different subjects were analyzed. In total, samples from 87 subjects were included in the analysis of all 18 BGMS. The specific subjects from whom samples were included in the analysis of the individual BGMS were different between BGMS. Reasons were, for example, systematically lower glucose concentrations for the whole blood–calibrated BGMS g, and data exclusions that did not affect all BGMS to the same degree. Data were excluded from analysis for the following reasons: (1) procedural error; (2) device deficiency; (3) BGMS provided no valid measurement result; (4) incomplete data set; (5) PO_2_ of adjusted samples <55 mm Hg (<7.3 kPa) or >85 mm Hg (>11.3 kPa) for glucose oxidase–based BGMS; (6) hemolysis in plasma samples for reference measurements; (7) quality control measurement results obtained with the reference measurement procedures before measuring blood samples were outside predefined limits; (8) difference between the first and second reference measurements exceeded the acceptance criteria for sample stability (as defined above); (9) mean reference measurement result outside the test system’s measurement range; (10) required number of samples in a BG concentration range already reached.

In this study, system accuracy was evaluated for each BGMS by comparison of its measurement results to results from the respective manufacturer’s reference measurement procedure according to labeling. Based on ISO 15197, the acceptability was determined by adding the relative number of results within ±15 mg/dL (0.83 mmol/L) for BG concentrations <100 mg/dL (5.55 mmol/L) to the number of results within ±15% for BG concentrations ≥100 mg/dL (5.55 mmol/L). More stringent criteria of ±10 mg/dL (0.56 mmol/L) and ±10%, and ±5 mg/dL (0.28 mmol/L) and ±5%, which ISO 15197 recommends to report, were applied as well.

In addition, the minimal deviation from the respective reference measurement procedure’s results within which ≥95% of results of the BGMS were found was calculated.

ISO 15197 additionally intends application of CEG analysis to three reagent system lots combined.^11^ For this analysis, the number and the percentage of results within the clinically acceptable CEG zones A and B were calculated for the evaluated reagent system lot.

Relative bias was calculated according to Bland and Altman.^22^

## Results

Statistical results are comprehensively presented in table 2. The minimal deviation from the manufacturer’s reference measurement procedure within which ≥95% of results of the respective BGMS were found was lowest for ContourNext One (±7.7 mg/dL (0.43 mmol/L) or ±7.7%) and highest for eBsensor (±19.7 mg/dL (1.09 mmol/L) or ±19.7%).

**Table 2:** Statistical results for the investigated test strip lot compared against the manufacturer’s reference measurement procedure: percentage of results within ISO 15197 accuracy criteria, minimal deviation from the reference measurement procedure’s results containing at least 95% of values and relative bias according to Bland and Altman (systems are ranked alphabetically)

#	BGMS	Reference measurement procedure	±15 mg/dL/±15%	±10 mg/dL/±10%	±5 mg/dL/±5%	Minimal deviation containing at least 95% of values	Relative bias	N
			%	%	%	mg/dL/%	%	
a	ABRA	GOD	91.0	71.0	36.5	±16.8	−2.9	200
b	Accu-Chek Guide	HK	100.0	97.5	82.0	±8.4	−1.6	200
c	AURUM	GOD	97.5	88.0	55.5	±13.5	2.6	200
d	CareSens Dual	GOD	96.5	84.5	48.0	±13.8	0.8	200
e	CERA-CHEK 1CODE	GOD	94.5	82.0	51.0	±15.2	−1.3	200
f	ContourNext One	GOD	100.0	99.5	77.0	±7.7	3.3	200
g	eBsensor	GOD	89.0	73.5	45.5	±19.7	3.9	200
h	FreeStyle Freedom Lite	GOD	99.5	91.5	46.5	±11.1	−6.6	200
i	GL50 evo	GOD	97.5	86.5	59.5	±13.4	1.7	200
j	GlucoCheck GOLD	GOD	100.0	92.0	54.5	±11.2	−4.5	200
k	GlucoMen areo 2K	GOD	98.0	86.0	49.0	±13.3	5.7	200
l	GluNEO	HK*	93.0	77.5	35.0	±16.2	−0.6	200
m	MyStar DoseCoach	GOD	95.0	85.0	56.0	±14.4	4.3	200
n	OneTouch Verio Flex	GOD	99.0	93.5	58.5	±11.9	4.2	200
o	Pic Gluco Test	GOD	98.0	87.5	54.5	±12.2	3.3	200
p	Rightest GM700S	GOD	99.5	96.0	67.0	±9.4	2.1	200
q	TRUEyou	GOD	99.0	85.5	56.0	±13.6	0.9	200
r	WaveSense JAZZ Wireless	GOD	95.0	84.0	55.0	±14.4	4.2	200

*No information about the manufacturer’s reference measurement procedure was available at the time of manuscript submission. Based on literature research^19^ and the investigator’s experience regarding reliability of measurement results, the HK-based procedure was assigned as primary reference measurement procedure for system accuracy evaluation.

GOD, glucose oxidase; HK, hexokinase.

**Figure 1:**
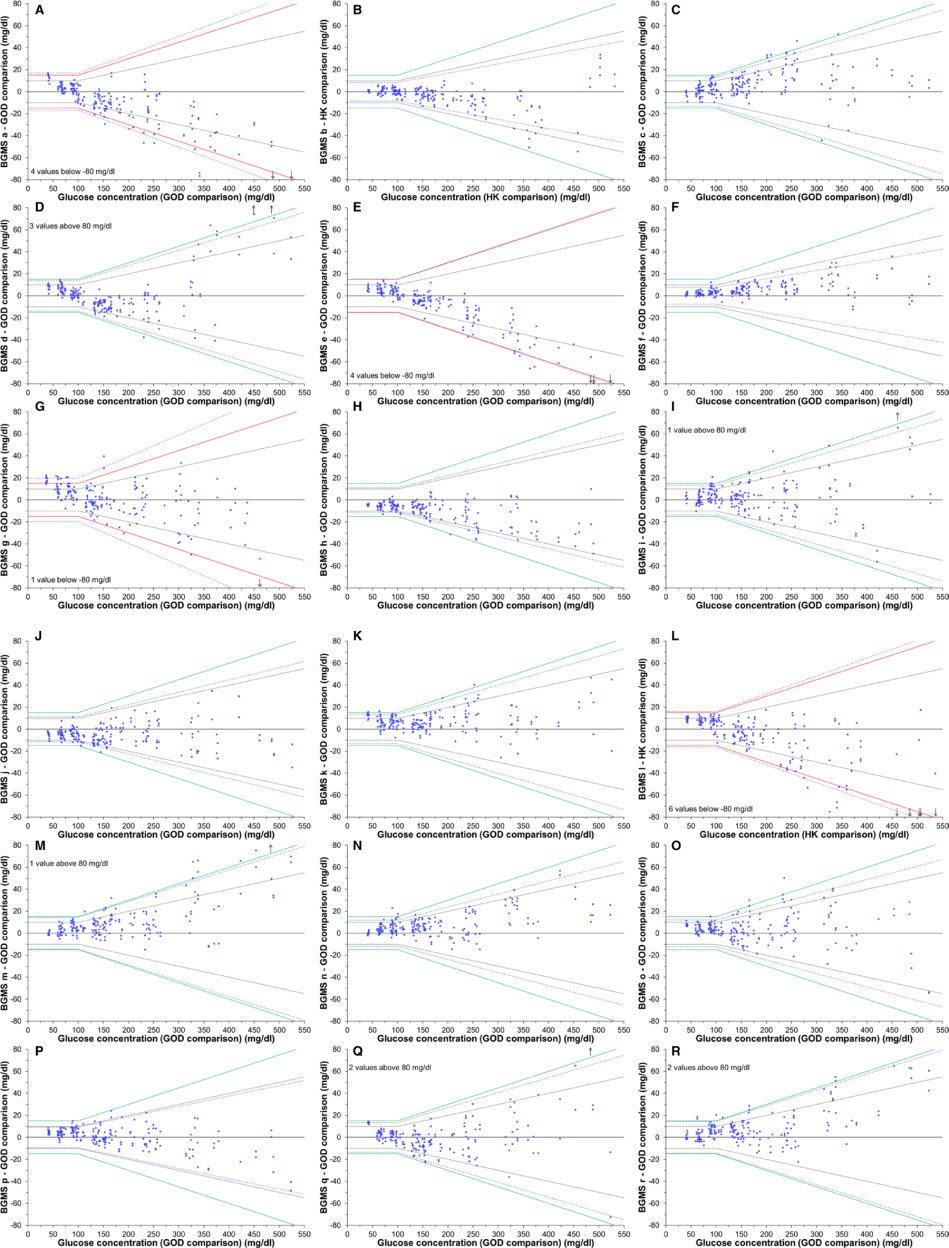
Difference plots for 18 blood glucose monitoring systems (BGMS) (n=200 for each difference plot) when evaluated against the manufacturer’s reference measurement procedure. Red/green solid lines: system accuracy criterion A in accordance to ISO 15197, ±15 mg/dL (0.83 mmol/L)/±15% (green lines were used if accuracy criterion a was fulfilled, red lines were used if accuracy criterion a was not fulfilled). Gray solid lines: accuracy limits of ±10 mg/dL (0.56 mmol/L)/±10%. Blue dashed lines: minimal deviation from the reference measurement procedure’s results within which ≥95% of results of the respective BGMS were found. For system l, no information about the manufacturer’s reference measurement procedure was available at the time of manuscript submission. Based on literature research^19^ and the investigator’s experience regarding reliability of measurement results, the hexokinase (HK)-based procedure was assigned as primary reference measurement procedure for system accuracy evaluation. If values were found outside the y-axis limits of ±80 mg/dL, a label was added to the upper or lower left corner of the BGMS’ subgraph and arrows indicate at which concentrations these values were found. GOD, glucose oxidase.

Minimum accuracy requirements as described above were fulfilled by 77.8% (14 out of 18) of the tested BGMS (table 2). The more stringent criterion of at least 95% of values within ±10 mg/dL (0.56 mmol/L) or ±10% of the respective reference measurement procedure’s results was achieved by 16.7% (3 out of 18) of the tested BGMS and none of the BGMS showed ≥95% of BG values within ±5 mg/dL (0.28 mmol/L) or ±5% (table 2). Percentages of results within ±15 mg/dL (0.83 mmol/L) or ±15% of the reference measurement procedure’s results ranged from 89.0% to 100.0% (figure 1A and B).

All BGMS showed 100% of results evaluable for CEG analysis (ie, BG values <550 mg/dL (30.52 mmol/L)) within the clinically acceptable zones A and B.

**Figure 2:**
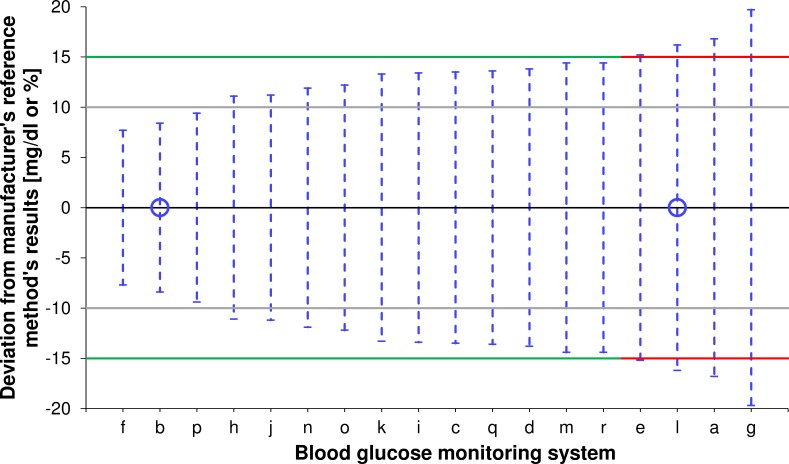
Minimal deviation of measurement results obtained with the tested blood glucose monitoring system (BGMS) (n=200 for each BGMS) from the respective manufacturer’s reference measurement procedure’s measurement results containing at least 95% of values (blue dashed lines; represented by blue funnels in figure 1A and B). For system l, no information about the manufacturer’s reference measurement procedure was available at the time of manuscript submission. Based on literature research^19^ and the investigator’s experience regarding reliability of measurement results, the hexokinase (HK)-based procedure was assigned as primary reference measurement procedure for system accuracy evaluation. Green/red solid lines: accuracy limits of ±15 mg/dL (0.83 mmol/L)/±15% (green lines were used if accuracy criterion a was fulfilled, red lines were used if accuracy criterion a was not fulfilled). Gray solid lines: accuracy limits of ±10 mg/dL (0.56 mmol/L)/±10%. BGMS with HK as manufacturer’s reference measurement procedure are marked with a blue open circle on the x-axis.

Figure 2 shows the ranking of all tested BGMS in regard of the minimal deviation from the manufacturers’ reference measurement procedures’ (table 1) results which allows the respective BGMS to just fulfill ISO 15197 accuracy criterion A with the evaluated reagent system lot.

## Conclusions

In this study, system accuracy of 18 CE-labeled, current-generation BGMS was evaluated with one reagent system lot each based on the international standard ISO 15197. The investigated reagent system lot of almost 80% of the investigated systems (14 out of 18) met ISO 15197 accuracy criteria when compared with the respective manufacturer’s reference measurement procedure (GOD or HK). All BGMS showed 100% of results within the clinically acceptable zones A and B of the CEG for the tested reagent system lot which indicate clinically accurate or acceptable measurement results.^23^

Regarding the interpretation of this study’s results, it must be taken into account that all results are based on one single reagent system lot per system and that variability between different reagent system lots is an important matter regarding system accuracy evaluation.^24^ Due to limited funding, testing three reagent system lots for each BGMS would only have been possible with a limited number of BGMS. Therefore, the decision was made to instead investigate only one reagent system lot, but for a larger number of BGMS. However, ISO 15197 demands that each of three reagent system lots that shall be tested in a system accuracy evaluation must pass accuracy criterion A on its own, whereas only criterion B has to be applied to all three reagent system lots together.^11^

Our finding of 14 BGMS passing criterion A with the tested reagent system lot leads to the conclusion that at least the other 4 (>20%) CE-labeled and currently available BGMS would not have fulfilled minimum accuracy requirements if the study were set up strictly according to ISO 15197 requirements (ie, if two additional reagent system lots per BGMS had been tested). This lack of accuracy is critical since diabetic patients often rely on BGMS accuracy and use BGMS measurement results for therapeutic decisions like administration of rescue carbohydrates or insulin dosing.^25^ For example, Breton and Kovatchev assessed the impact of BGMS measurement accuracy on four scenarios in diabetes therapy in an in silico modeling study.^26^ They found that the probability to measure BGMS results >70 mg/dL (3.88 mmol/L) with a “true” glucose concentration of 60 mg/dL (3.33 mmol/L) increased 10-fold when the allowed range within which 95% of results were found is increased from ±7 mg/dL (0.39 mmol/L) or ±10% (for glucose concentrations <75 mg/dL (4.16 mmol/L) or ≥75 mg/dL (4.16 mmol/L), respectively) to ±15 mg/dL (0.83 mmol/L) or ±20%. Furthermore, increasingly inaccurate BGMS results were found to lead to higher glycemic variability when pre-meal and insulin correction boluses were integrated in the model. In their final scenario, Breton and Kovatchev found that HbA1c progressively increased when the allowed range within which 95% of results were found exceeded ±4 mg/dL (0.22 mmol/L) or ±5% (for glucose concentrations <75 mg/dL (4.16 mmol/L) or ≥75 mg/dL (4.16 mmol/L), respectively).

In addition, most continuous glucose monitoring (CGM) systems are regularly calibrated with BGMS values.^27^ In 2019, an international consensus group specified recommendations on times that should be spent in predefined glucose ranges.^28^ However, in manually calibrated CGM systems, the quality of CGM values obtained depends on the quality of the calibration value. Thus, even if the targeted time in range is reached, diabetes therapy might not be optimally adjusted to an individual’s personal needs when therapy decisions are based on CGM values and CGM calibration was performed with an inaccurate BGMS.

Furthermore, falsely elevated measurement results may keep dangerous hypoglycemic metabolic states hidden or they could lead to inadequately large insulin doses. Falsely lowered measurement results might result in the reverse scenario as a patient with diabetes might consume readily absorbable carbohydrates, and thus such measurements could lead to long-term high blood glucose levels subsequently resulting in elevated HbA_1c_ levels and the risk of associated long-term complications.^25^

Comparable studies within the recent years (BGMS purchased on the market; procedures and evaluation based on ISO 15197) with the aim of giving a comprehensive overview of BGMS accuracy showed percentages of BGMS not meeting accuracy requirements of ISO 15197 ranging from 30% to 79%.^15 29 30^ A multicenter accuracy study evaluating one system reagent lot for each of 19 BGMS manufactured in the Asia-Pacific region showed ≈79% (15 out of 19) of BGMS not meeting the accuracy requirements of ISO 15197. However, to minimize variance in reference measurements, YSI 2300 STAT Plus was the only reference measurement procedure used.^30^ In a study by Freckmann *et al* 15 with three tested reagent system lots per BGMS in 2015, 33% (3 out of 9) BGMS did not meet ISO 15197 accuracy requirements. In another accuracy study with the 18 best-selling BGMS in the USA by Klonoff *et al*,^29^ 12 out of 18 tested BGMS were not meeting accuracy requirements similar to ISO 15197. However, comparability of the study by Klonoff *et al* and our study is limited because Klonoff included BGMS which were cleared by the Food and Drug Administration (FDA) when accuracy criteria were milder than current criteria and applied a non-official accuracy standard which is more lenient than ISO and FDA specifications.^29^

As highlighted by King *et al*, many published BGMS accuracy studies are not performed independently from the manufacturer, and BGMS tend to perform better in manufacturer-supported studies.^31^ This raises the question whether there is a relevant publication bias, implying that a smaller number of BGMS than gathered from the literature might actually provide adequate measurement accuracy. In that context, this study’s strengths are reflected by the funding from six different parties who were not involved regarding study procedures, by the manufacturer-independent BGMS acquisition and study conduct, and by the BGMS selection of only current-generation systems with focus on market relevance.

A limiting aspect of this study is that glucose measurements in a controlled laboratory environment by healthcare professionals or well-trained study personnel, as outlined by ISO 15197 clause 6.3, tend to lead to more accurate results than lay-user measurements.^32 33^ Deviations between experts’ measurement results and those of lay-users can be traceable to device specific requirements (eg, insufficient blood volume)^34^ and/or to non–device-specific sources of error (eg, contamination of puncture site with glucose-containing substances).^35 36^

A weakness of the current regulatory framework is that an independent and regular assessment of measurement accuracy is not mandatory for market approval of BGMS and that the fulfillment of the requirements to obtain the CE mark has to be verified just one single time for that purpose.^37^ Furthermore, according to the FDA, some manufacturers or testing institutions commissioned by these manufacturers might be tempted to falsify data for market approval.^38^

Generally, this and other studies as well have shown that the lack of independent post-market BGMS testing allows CE-labeled systems which do not reliably fulfill ISO 15197 criteria to enter the market and that there is a considerable variation in measurement accuracy even among BGMS fulfilling these criteria.^12 13 29 30 39^ Independent testing and regular post-market surveillance of all available BGMS, not just for modern systems but also for older generations of BGMS, which still are on the market, therefore is necessary to allow patients with diabetes and healthcare professionals selecting a BGMS that is best possible for diabetes therapy.
