# Supplementary material for: System accuracy evaluation of 18 CE-marked current-generation blood glucose monitoring systems based on EN ISO 15197:2015
Source: BMJ Open Diabetes Res Care. 2020 Jan 15;8(1):e001067. doi: 10.1136/bmjdrc-2019-001067 (PMC7039612; doi:10.1136/bmjdrc-2019-001067)
Supplement: Supplementary data [file bmjdrc-2019-001067supp001.pdf]

Supplementary Material

Table 1: Serial numbers of all tested meters used in the study.

#	Meter number	Serial number	#	Meter number	Serial number
a	1	M17KL067228	j	1	4289118200036703
	2	M17KL066775		2	4289118200036613
b	1	92107098575	k	1	GT269392
	2	92107165698		2	GT269359
c	1	4141118100088986	l	1	G101B18C2701669
	2	414111810008871D		2	G101B18C2701670
d	1	F024096G0080	m	1	MHAA10XH401914
	2	F024096G0022		2	MHAA10XH102343
e	1	G467J2005345	n	1	ZFLGD6DW
	2	G467J2005220		2	ZFLGD10H
f	1	6113951	o	1	M15D03AC1118
	2	6113953		2	M15D03AC1125
g	1	eB-G2Z0B1G60195	p	1	2780QBA7949
	2	eB-G2Z0B1G60206		2	2780QBA7948
h	1	CEGY136-M0424	q	1	MY0260549
	2	CEGX256-M1145		2	MY0260568
i	1	D15/011381	r	1	(21)DBDD03YH203261
	2	D15/011382		2	(21)DBDD28XH601356

Pleus S, *et al. BMJ Open Diab Res Care* 2020; 8:e001067. doi: 10.1136/bmjdrc-2019-001067
